# A first-in-human study of AMG 208, an oral MET inhibitor, in adult patients with advanced solid tumors

**DOI:** 10.18632/oncotarget.4472

**Published:** 2015-06-19

**Authors:** David S. Hong, Peter Rosen, A. Craig Lockhart, Siqing Fu, Filip Janku, Razelle Kurzrock, Rabia Khan, Benny Amore, Isaac Caudillo, Hongjie Deng, Yuying C. Hwang, Robert Loberg, Gataree Ngarmchamnanrith, Darrin M. Beaupre, Peter Lee

**Affiliations:** ^1^ MD Anderson Cancer Center, Houston, TX, USA; ^2^ Tower Cancer Research Foundation, Beverly Hills, CA, USA; ^3^ Washington University School of Medicine, St. Louis, MO, USA; ^4^ Amgen Inc., Seattle, WA, USA; ^5^ Amgen Inc., Thousand Oaks, CA, USA

**Keywords:** MET, first-in-human, solid tumors, prostate cancer, small molecule

## Abstract

**Background:**

This first-in-human study evaluated AMG 208, a small-molecule MET inhibitor, in patients with advanced solid tumors.

**Methods:**

Three to nine patients were enrolled into one of seven AMG 208 dose cohorts (25, 50, 100, 150, 200, 300, and 400 mg). Patients received AMG 208 orally on days 1 and days 4–28 once daily. The primary objectives were to evaluate the safety, tolerability, pharmacokinetics, and maximum tolerated dose (MTD) of AMG 208.

**Results:**

Fifty-four patients were enrolled. Six dose-limiting toxicities were observed: grade 3 increased aspartate aminotransferase (200 mg), grade 3 thrombocytopenia (200 mg), grade 4 acute myocardial infarction (300 mg), grade 3 prolonged QT (300 mg), and two cases of grade 3 hypertension (400 mg). The MTD was not reached. The most frequent grade ≥3 treatment-related adverse event was anemia (*n* = 3) followed by hypertension, prolonged QT, and thrombocytopenia (two patients each). AMG 208 exposure increased linearly with dose; mean plasma half-life estimates were 21.4–68.7 hours. One complete response (prostate cancer) and three partial responses (two in prostate cancer, one in kidney cancer) were observed.

**Conclusions:**

In this study, AMG 208 had manageable toxicities and showed evidence of antitumor activity, particularly in prostate cancer.

## INTRODUCTION

The receptor tyrosine kinase MET mediates multiple cellular processes, including proliferation, survival, migration, and invasion in normal and tumor cells [1-3]. MET can be activated through various mechanisms, such as ligand-dependent activation through binding of hepatocyte growth factor (HGF), and ligand-independent activation through overexpression, gene amplification, and activating mutations [1]. MET is often dysregulated in various cancers, including lymphoma, melanoma, gastric, lung, colorectal, head and neck, renal, and ovarian [4-11], providing a strong rationale for targeting MET [1-3]. Elevated MET expression has been correlated with poor prognosis [7], and *MET* amplification has been associated with drug resistance to epidermal growth factor receptor (EGFR) inhibitors [12, 13]. Preclinical data suggest that concurrently inhibiting the MET and vascular endothelial growth factor (VEGF) pathways has synergistic effects [14].

AMG 208 is a small-molecule MET inhibitor with a 50% inhibitory concentration (IC_50_) against wild-type MET of 5.2 nM. At higher concentrations, AMG 208 inhibited other kinases, such as VEGF receptor 2 (VEGF-R2, IC_50_ = 112 nM; data on file). AMG 208 suppressed proliferation and induced apoptosis in human tumor xenograft models (data on file).

We conducted a first-in-human study of AMG 208 to investigate its safety, tolerability, pharmacokinetics, and pharmacodynamics in patients with advanced solid tumors (ClinicalTrials.gov identifier: NCT00813384). We also evaluated antitumor activity and MET expression, amplification, and mutation status as potential biomarkers of response.

## RESULTS

### Patient characteristics and disposition

Fifty-four patients were enrolled and received ≥1 dose of AMG 208: 25 mg (*n* = 6), 50 mg (*n* = 4), 100 mg (*n* = 4), 150 mg (*n* = 3), 200 mg (*n* = 16), 300 mg (*n* = 10), and 400 (*n* = 11). The first patient enrolled on December 29, 2008, and the last patient completed the study on July 25, 2012. In the 25-mg cohort, the first three patients enrolled were not evaluable (did not complete the dose-limiting toxicity [DLT] assessment period defined as the first 28 days of treatment), so three additional patients were enrolled. Table 1 summarizes demographics and baseline characteristics. The most common primary tumor types were prostate (18.5%) followed by colon (11.1%), esophageal (11.1%), and non-small cell lung cancer (NSCLC, 11.1%). Reasons for discontinuing AMG 208 treatment included disease progression (64.8%), adverse events (AEs, 16.7%), withdrawal of partial consent (5.6%), and requirement for alternative therapy (3.7%). The median number of AMG 208 doses received per patient was 27 (range, 1–671) and was highest in the 400-mg cohort (110; range, 9–306). Three (5.6%) patients had dose reductions.

**Table 1:** Demographics and baseline characteristics

	All Patients (N = 54)
Sex, n (%)	
Male	36 (66.7)
Female	18 (33.3)
Race, n (%)	
White/Caucasian	39 (72.2)
Hispanic/Latino	7 (13.0)
Black/African American	5 (9.3)
Asian	3 (5.6)
Age, median (range), y	60.5 (39­–80)
ECOG performance status at baseline, n (%)	
0	28 (52)
1	26 (48)
Disease stage, n (%)	
II	1 (2)
III	4 (7)
IV	48 (89)
Unknown	1 (2)
Primary tumor type, n (%)	
Prostate	10 (18.5)
Colon	6 (11.1)
Esophageal	6 (11.1)
Non-small cell lung	6 (11.1)
Kidney	5 (9.3)
Head and neck squamous cell carcinoma	3 (5.6)
Bladder	2 (3.7)
Carcinoma of unknown origin	2 (3.7)
Malignant melanoma	2 (3.7)
Ovarian	2 (3.7)
Stomach	1 (1.9)
Cervix	1 (1.9)
Oral	1 (1.9)
Pancreas	1 (1.9)
Soft tissue sarcoma	1 (1.9)
Uterine	1 (1.9)
Other^a^	4 (7.4)
Prior radiotherapy, n (%)	32 (59.3)
Number of prior chemotherapy regimens, n (%)	
1	4 (7)
2	15 (28)
≥3	35 (65)

a Includes adenoid cystic carcinoma, appendiceal adenocarcinoma, metastatic insular thyroid cancer, and poorly differentiated adenocarcinoma of the gallbladder. ECOG, Eastern Cooperative Oncology Group.

### Safety and tolerability

**Figure 1:**
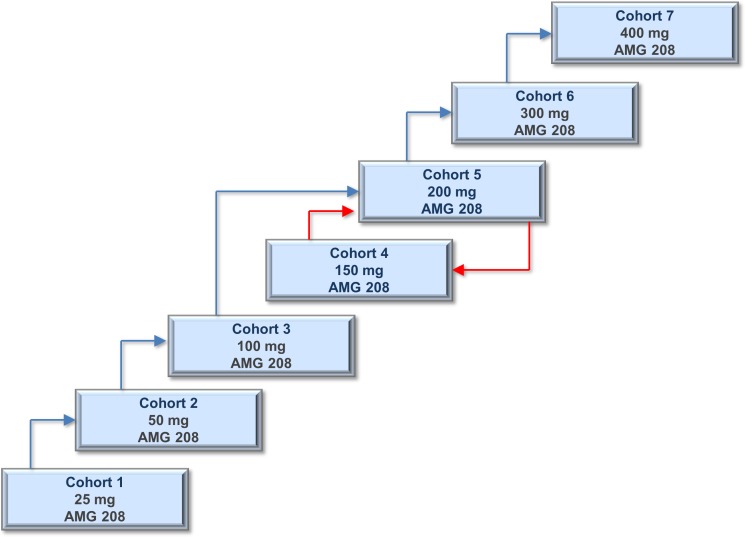
Schematic overview of the dose escalation cohorts Three to nine patients were enrolled into one of the following seven AMG 208 dose cohorts: 25, 50, 100, 150, 200, 300, and 400 mg. A standard 3+3 design was followed in cohorts 1–3, and a modified 3+3+3 design was followed in cohorts 4–7. The protocol was amended to evaluate an intermediate dose level of 150 mg after two DLTs (out of six patients) were observed with 200 mg AMG 208 (red arrow); re-escalation to 200 mg occurred after the 150-mg dose cohort was considered well tolerated (red arrow).

Six patients had DLTs: 200 mg (*n* = 2), 300 mg (*n* = 2), and 400 mg (*n* = 2). In the 200-mg cohort, seven patients were initially enrolled, of whom two had a DLT (grade 3 increased aspartate aminotransferase [AST] and grade 3 thrombocytopenia), four completed without a DLT, and one withdrew early from the study due to disease progression. The protocol was amended to de-escalate to 150 mg and then re-escalate to 200 mg (Figure 1); three additional patients were then enrolled to the 200-mg cohort, of whom two completed without a DLT, and one withdrew from the study due to stroke, which was not considered related to AMG 208. The 200-mg cohort was expanded to four additional patients, of whom three completed without a DLT, and one withdrew early from the study. Per protocol, two additional patients were allowed to enroll to the 200-mg cohort because of MET-positive status. In the 300-mg cohort, seven patients were initially enrolled, of whom two had a DLT (grade 4 acute myocardial infarction and grade 3 prolonged QT), four completed without DLT, and one withdrew early from the study. Three additional patients were enrolled to the 300-mg cohort, and these three completed without a DLT. In the 400-mg cohort, eight patients were initially enrolled, of whom two had a DLT (both grade 3 hypertension), four completed without a DLT, and two withdrew early from the study. Three additional patients were enrolled to the 400-mg cohort, and these three completed without a DLT. A maximum tolerated dose (MTD) was not determined.

Forty-five patients had treatment-related AEs, the most common being fatigue (44.4%) and nausea (33.3%) (Table 2). Thirteen patients reported grade ≥3 treatment-related AEs, with anemia (5.6%), hypertension (3.7%), prolonged QT (3.7%), and thrombocytopenia (3.7%) being the most frequently reported (Table 2).

**Table 2:** Treatment-related adverse events

		AMG 208 Dose Cohort (mg)						
	All Patients (N=54)	25 mg (n=6)	50 mg (n=4)	100 mg (n=4)	150 mg (n=3)	200 mg (n=16)	300 mg (n=10)	400 mg (n=11)
**Patients with any grade AEs, n (%)**	45 (83.3)	2 (33.3)	3 (75.0)	4 (100.0)	3 (100.0)	14 (87.5)	8 (80.0)	11 (100.0)
Fatigue	24 (44.4)	2 (33.3)	2 (50.0)	2 (50.0)	3 (100.0)	3 (18.8)	4 (40.0)	8 (72.7)
Nausea	18 (33.3)	1 (16.7)	2 (50.0)	0 (0)	1 (33.3)	2 (12.5)	4 (40.0)	8 (72.7)
Hypertension	12 (22.2)	0	0	1 (25.0)	0	6 (37.5)	2 (20.0)	3 (27.3)
Diarrhea	11 (20.4)	0	0	0	0	3 (18.8)	2 (20.0)	6 (54.5)
Anemia	10 (18.5)	0	0	2 (50.0)	2 (66.7)	4 (25.0)	1 (10.0)	1 (9.1)
Increased AST	9 (16.7)	0	0	0	2 (66.7)	4 (25.0)	0	3 (27.3)
Decreased appetite	9 (16.7)	0	1 (25.0)	0	0	2 (12.5)	3 (30.0)	3 (27.3)
Leukopenia	9 (16.7)	0	0	0	3 (100.0)	3 (18.8)	1 (10.0)	2 (18.2)
Increased ALT	8 (14.8)	0	0	0	1 (33.3)	4 (25.0)	0	3 (27.3)
Achromotrichia acquired	7 (13.0)	0	0	0	0	2 (12.5)	2 (20.0)	3 (27.3)
Thrombocytopenia	7 (13.0)	0	0	0	1 (33.3)	2 (12.5)	1 (10.0)	3 (27.3)
Vomiting	6 (11.1)	0	1 (25.0)	0	0	0	1 (10.0)	4 (36.4)
Increased blood creatinine	5 (9.3)	0	0	0	1 (33.3)	3 (18.8)	1 (10.0)	0
Hypomagnesemia	5 (9.3)	0	0	1 (25.0)	0	3 (18.8)	0	1 (9.1)
**Patients with grade ≥3 AEs, n (%)**	13 (24.1)	0	0	1 (25.0)	0	3 (18.8)	3 (30.0)	6 (54.5)
Anemia	3 (5.6)	0	0	1 (25.0)	0	1 (6.3)	1 (10.0)	0
Hypertension	2 (3.7)	0	0	0	0	0	0	2 (18.2)
Prolonged electrocardiogram QT	2 (3.7)	0	0	0	0	0	1 (10.0)	1 (9.1)
Thrombocytopenia	2 (3.7)	0	0	0	0	1 (6.3)	0	1 (9.1)
Acute myocardial infarction	1 (1.9)	0	0	0	0	0	1 (10.0)	0
Increased AST	1 (1.9)	0	0	0	0	1 (6.3)	0	0
Increased blood creatinine	1 (1.9)	0	0	0	0	1 (6.3)	0	0
Increased blood creatinine phosphokinase	1 (1.9)	0	0	0	0	0	0	1 (9.1)
Hyperglycemia	1 (1.9)	0	0	0	0	0	0	1 (9.1)
Neutropenia	1 (1.9)	0	0	0	0	0	0	1 (9.1)
Decreased neutrophil count	1 (1.9)	0	0	0	0	0	0	1 (9.1)
Pulmonary embolism	1 (1.9)	0	0	0	0	0	0	1 (9.1)

Any grade treatment-related AEs occurring in ≥5 patients overall and any grade ≥3 treatment-related AEs are shown. AEs were coded using MedDRA version 15.0. AE, adverse event; ALT, alanine aminotransferase; AST, aspartate aminotransferase.

Seven patients reported treatment-related AEs considered serious: 200 mg (*n* = 3; increased AST, thrombocytopenia, and increased serum creatinine), 300 mg (*n* = 1; acute myocardial infarction), and 400 mg (*n* = 3; hypertension, pulmonary embolism, and neutropenia). Of these seven patients, three had treatment-related serious AEs leading to AMG 208 discontinuation: 200 mg (*n* = 1; increased AST), 300 mg (*n* = 1; acute myocardial infarction), and 400 mg (*n* = 1; hypertension). The increased AST was observed in a 43-year-old female with stage IV papillary renal cell carcinoma who developed grade 3 elevation of AST with grade 1 elevation of alanine aminotransferase (ALT) on day 8 of the study. AMG 208 was discontinued, and the AST and ALT were normalized within 1 week. The investigator did not report any relevant concomitant medications, and there were no reports of other liver function test abnormalities. The acute myocardial infarction was observed in a 67-year-old male with stage IV NSCLC who had a history of hypertension, hyperlipidemia, cerebrovascular accident, and coronary artery disease. The patient reported chest pain with associated shortness of breath 10 and 14 days after receiving the first dose of AMG 208, and he was diagnosed with ST elevation myocardial infarction. Cardiac catheterization revealed ulcerated plaques in the right coronary artery and moderate/diffuse disease of the left anterior descending and circumflex arteries. The hypertension was observed in a 76-year-old male with stage IV prostate cancer who had a history of controlled hypertension. His blood pressure was elevated on days 7 to 10 of the study and ranged from 178/88 to 210/90. There were no other predisposing risk factors for elevated blood pressure except for the presence of bone pain before and during the event. AMG 208 was discontinued, and the event resolved within 4 days of discontinuation.

Five patients died during the study, and none were considered treatment related (disease progression, *n* = 4; pulmonary hemorrhage, *n* = 1). The patient with pulmonary hemorrhage was diagnosed with pulmonary hypertension and pulmonary aspergillosis and had clear evidence of metastatic disease in the lung; thus, the grade 5 pulmonary hemorrhage was considered related to the disease.

### Pharmacokinetics

**Figure 2:**
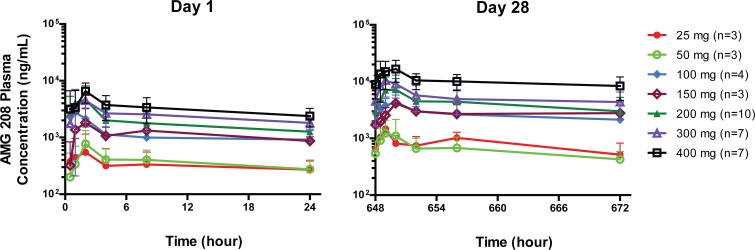
Plasma concentration time profiles of AMG 208 on days 1 and 28 following oral administration on days 1 and 4 to 28 once daily Data points represent means ± standard deviations.

AMG 208 pharmacokinetics was estimated for 53 patients who received ≥1 dose of AMG 208. After oral administration, linear increases of approximately 10- to 12-fold were observed over the 25- to 400-mg dose range in maximum concentration (C_max_) and area under the concentration-time curve from time 0 to 24 hours (AUC_0–24h_) exposures. In the 400-mg cohort, mean C_max_ and AUC_0–24h_ exposures on day 28 were 18.4 μg/mL and 245 μg•h/mL, respectively. Mean plasma concentration-time profiles from each cohort are shown for 36 patients who completed 28 days of AMG 208 dosing (Figure 2). AMG 208 mean plasma half-life estimates ranged from 21.4 to 68.7 hours and were consistent with a 1.81- to 3.43-fold accumulation observed after 28 days of repeated dosing.

### Pharmacodynamics

**Figure 3:**
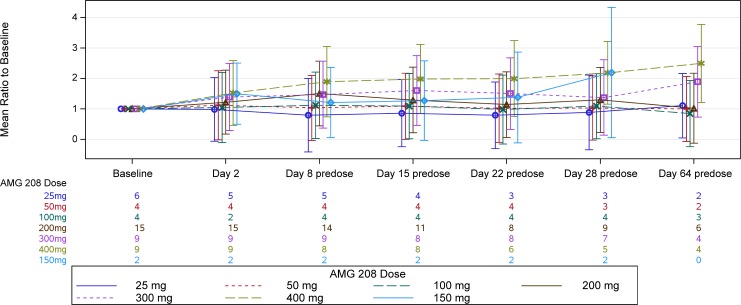
PlGF mean ratio to baseline by treatment arm Patients who received ≥1 dose of AMG 208 and had a measurable baseline concentration of PlGF were analyzed. The mean log ratio to baseline and standard error were computed, and the results were anti-logged and presented as mean ratio to baseline. Results from day 64 were excluded due to small sample size.

The pharmacodynamic effects of AMG 208 on the following circulating biomarkers were evaluated: soluble MET, HGF, placental growth factor (PlGF), VEGF-R2, c-Kit, sFlt-1, VEGF, serum C-terminal telopeptide of type 1 collagen (sCTx), type 1 procollagen N-terminal propeptide (P1NP), and bone alkaline phosphatase (BALP). PlGF demonstrated a pharmacodynamic response to AMG 208; mean PlGF levels increased the most with the 400-mg dose at all time points (Figure 3). No other circulating biomarkers demonstrated a pharmacodynamic effect.

### Antitumor activity

**Figure 4:**
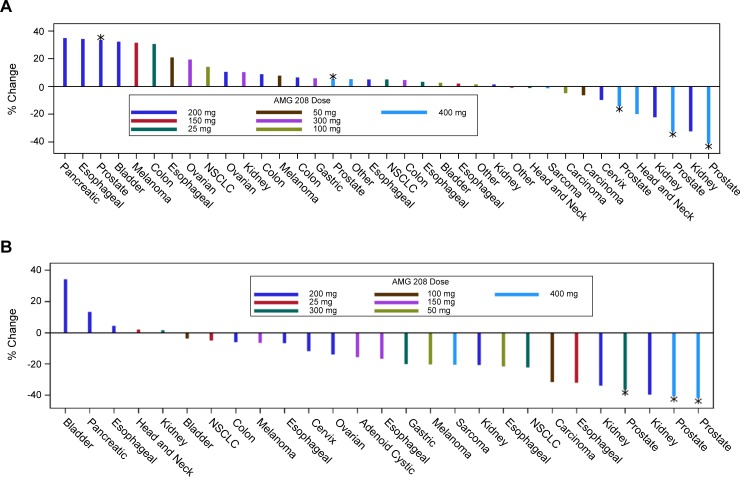
Antitumor activity of AMG 208 **A.** The percent change in the sum of the longest diameter (SLD) for the best postdose response is shown. Patients with baseline and ≥1 post-baseline SLD for the target lesion were analyzed. Thirty-seven patients are shown, and 17 were not included because of the following reasons: five patients were evaluated with nontarget lesions only (four prostate and one NSCLC), one patient with NSCLC had progressive disease due to a new lesion, and 11 patients did not have baseline and/or post-baseline scans. **B.** The percent change in the sum of ^18^F-FLT SUV_max_ (1 cm spot) at week 5 day 29. Only patients with both baseline and week 5 day 29 SUV_max_ (1 cm spot) are shown. *Prostate cancer. ^†^Carcinoma of unknown origin. NSCLC, non-small cell lung cancer; SUV_max_, maximum standardized uptake value.

**Figure 5:**
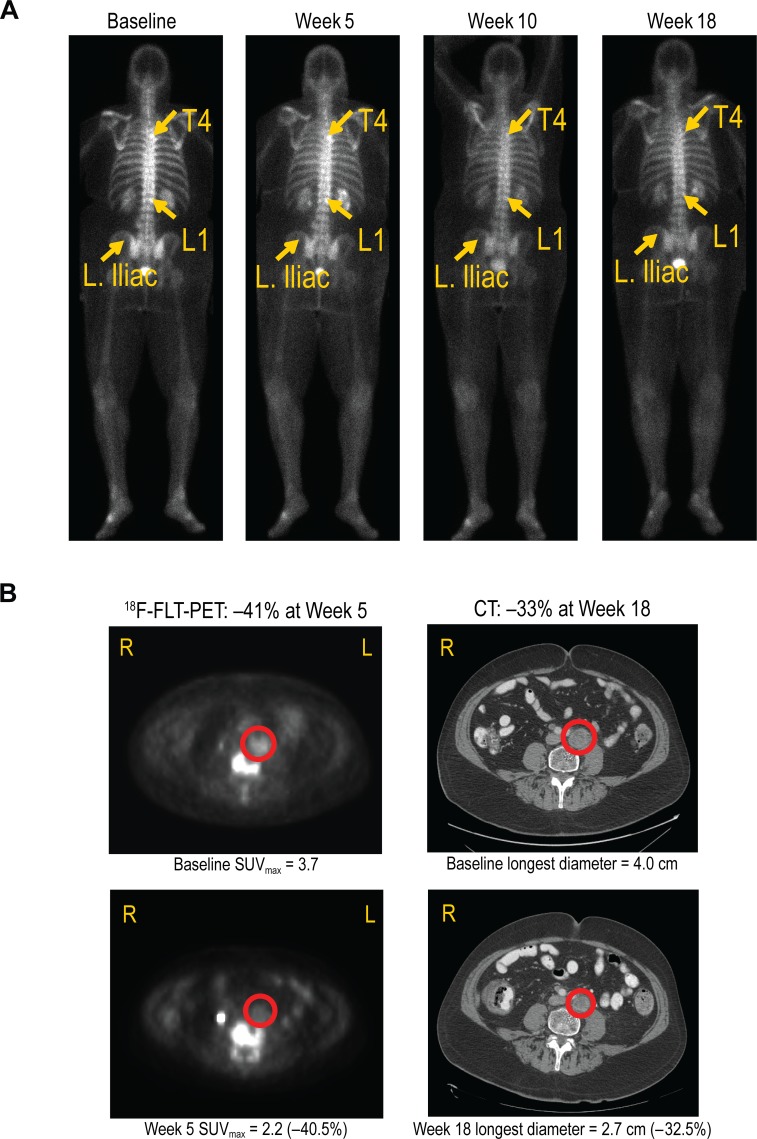
**A.** Complete response in a 66-year-old patient with prostate cancer treated with 300 mg AMG 208 (bone scans). At baseline, bone metastasis was present at T4 and L1. At week 18, evidence of bone metastasis was not observed. **B.** Partial response in a 63-year-old patient with prostate cancer treated with 400 mg AMG 208 (^18^F-FLT-PET and CT scans).

Forty-three patients had responses evaluated at the sites based on computed tomography (CT) and/or bone scans using the modified Response Evaluation Criteria for Solid Tumors (RECIST) version 1.0. Eleven patients went off study early (due to disease progression [*n* = 3], DLT [*n* = 3], AE [*n* = 3], partial consent withdrawn [n = 1], or ineligibility determined after first dose [*n* = 1]) and had no follow-up scans for response evaluation. Figure 4A shows the change in the sum of the longest diameter for the best postdose response. There were one complete response (CR) and three partial responses (PRs). The CR was observed in a 66-year-old patient with prostate cancer at week 18, based on nontarget lesions evaluated by bone scans (300-mg cohort, Figure 5A); this patient was on the study for approximately 57 weeks. One confirmed PR was observed in a patient with kidney cancer at week 9 (200-mg cohort); this patient had a 33% tumor reduction and was on the study for approximately 23 weeks. Two unconfirmed PRs were observed in two patients with prostate cancer (both 400-mg cohort). One patient had a 33% tumor reduction and was on the study for approximately 31 weeks. The second patient had a 41% tumor reduction and was on the study for approximately 35 weeks. Twenty-eight patients had stable disease, and 11 patients had progressive disease.

Forty-two patients had responses evaluated by independent central review based on CT and/or bone scans. Eleven patients went off study early and had no follow-up scans, and one patient had incomplete coverage of target lesion at the follow-up scan and could not be evaluated. Thirty-three patients had stable disease, and nine patients had progressive disease.

Antitumor activity was further evaluated by 3′-deoxy-3′-^18^F-fluorothymidine (^18^F-FLT) positron emission tomography (PET) scans (Figure 4B). Twenty-seven patients had evaluable baseline and week 5 ^18^F-FLT-PET scans. Seven patients had ≥25% reduction from baseline in ^18^F-FLT uptake (maximum standardized uptake value [SUV_max_]) at week 5 day 29, indicating a treatment response in tumor cell proliferation. Two of the seven proliferative responders (1 patient with prostate cancer (Figure 5B) and 1 patient with kidney cancer) also had PR by RECIST 1.0 (33% reduction based on site read).

Of the 10 patients with prostate cancer, three patients had a response per modified RECIST 1.0. As mentioned earlier, one patient with prostate cancer had a CR on bone scan (300-mg cohort). Two patients with prostate cancer had unconfirmed PRs based on CT scans, both in the 400-mg cohort. One patient had a 33% tumor reduction at week 17. The second patient had a 41% tumor reduction at week 27. Furthermore, three patients with prostate cancer were proliferative responders (36%, 41%, and 42% reductions in ^18^F-FLT uptake).

### Biomarkers

Twenty-two, 29, and 25 patients were analyzed for MET by immunohistochemistry (IHC), fluorescence in situ hybridization (FISH), and sequencing, respectively. No associations between response and MET testing were observed (Table 3).

**Table 3:** Tumor response by MET analysis

							Cytoplasmic IHC			Membrane IHC		
Dose (mg)	Primary Tumor Type	Best Tumor Response	Best % Change in SLDa	% Change in Sum of SUVmaxb	Sequencing	FISH	% Pos	H-score	MAX SI	% Pos	H-score	MAX SI
25	Colon	PD	30.65	-	M	Neg	90	90	1+	0	0	0
	Esophageal	SD	3.39	−32.00	ND	ND	100	200	3+	1	1	1+
	NSCLC	SD	5.00	−4.94	M	Neg	10	10	1+	50	50	1+
50	Esophageal	PD	20.97	−21.55	W	Neg	80	80	1+	60	70	2+
	Carcinoma of unknown origin	SD	−6.45	-	W	Neg	0	0	0	0	0	0
100	Appendiceal adenocarcinoma	SD	1.39	-	W	Neg	95	155	2+	10	10	1+
	Bladder	SD	2.63	−3.70	F	Neg	0	0	0	0	0	0
	Carcinoma of unknown origin	SD	−5.02	−31.68	W	Neg	1	1	1+	0	0	0
	NSCLC	SD	14.05	-	F	ND	15	15	1+	0	0	0
150	Esophageal	PD	2.02	−16.67	W	ND	ND	ND	ND	ND	ND	ND
	Melanoma	PD	31.43	−6.57	W	Neg	0	0	0	0	0	0
200	Kidney	PR	−32.51	−39.68	ND	ND	95	105	2+	40	70	3+
	Ovarian	SD	10.56	−13.98	ND	Neg	60	60	1+	0	0	0
	Esophageal	SD	5.13	−6.67	ND	Neg	95	145	2+	60	70	2+
	Colon	SD	6.45	−6.06	ND	Neg	99	109	2+	20	20	1+
	Prostate	PD	33.33	−	F	ND	ND	ND	ND	ND	ND	ND
	Colon	PD	8.92	−	W	Neg	80	85	2+	10	10	1+
	Esophageal	PD	34.37	4.38	F	ND	25	30	2+	1	1	1+
	Kidney	SD	−22.22	−33.75	ND	Neg	95	137	3+	63	67	3+
	Bladder	PD	32.35	34.26	F	Neg	10	11	2+	10	16	3+
300	Prostate	CR	-	-	W	Neg	ND	ND	ND	ND	ND	ND
	Kidney	PD	10.32	1.49	M	Neg	ND	ND	ND	ND	ND	ND
	NSCLC	SD	-	−22.22	W	ND	ND	ND	ND	ND	ND	ND
	Stomach	SD	5.94	−20.19	W	Neg	ND	ND	ND	ND	ND	ND
	Prostate	SD	-	−36.40	W	Neg	ND	ND	ND	ND	ND	ND
	Prostate	SD	-	-	W	Neg	ND	ND	ND	ND	ND	ND
	Ovarian	SD	19.35	-	W	Neg	ND	ND	ND	ND	ND	ND
400	Prostate	PR	−32.50	−40.54	F	ND	ND	ND	ND	ND	ND	ND
	Head and neck squamous cell carcinoma	SD	−20.00	-	F	ND	ND	ND	ND	ND	ND	ND
	Prostate	SD	5.19	−41.83	ND	Neg	ND	ND	ND	ND	ND	ND
	Prostate	SD	−14.29	-	W	Pos	ND	ND	ND	ND	ND	ND
	Prostate	PR	−41.18	-	ND	Neg	ND	ND	ND	ND	ND	ND
	Poorly differentiated adenocarcinoma of gallbladder	SD	5.17	-	W	Neg	ND	ND	ND	ND	ND	ND

Patients with available data for MET biomarker testing and evaluable best tumor response (site reads), percent change in SLD (site reads), or percent change in sum of SUVmax are shown. aFrom baseline. b1 cm spot; week 5 day 29. F, failed; M, mutated; MAX SI, maximum staining intensity; SD, stable disease; SLD, sum of the longest diameter; ND, not determined; Neg, negative; PD, progressive disease; Pos, positive; PR, partial response; W, wild type.

## DISCUSSION

In this first-in-human study of AMG 208, an oral MET inhibitor, the MTD was not reached at the highest dose tested (400 mg), and AMG 208 had manageable toxicities. The most common any grade AEs included fatigue and nausea; the most common grade ≥3 AE was anemia. DLTs were reported in six patients at the 200- to 400-mg dose levels.

Similar toxicities were observed with AMG 208 as seen with other multikinase VEGF/MET inhibitors. In clinical studies of cabozantinib, a multikinase MET and VEGF inhibitor, common AEs included fatigue, decreased appetite, and diarrhea [15, 16], AEs also observed with AMG 208. Moreover, in a phase 2 study, foretinib, a dual MET/VEGF-R2 inhibitor, had a similar toxicity profile to AMG 208; common AEs included fatigue, hypertension, and gastrointestinal toxicities [17].

AMG 208 was orally bioavailable at the doses tested and exhibited a favorable pharmacokinetic profile. AMG 208 exposures increased linearly up to the 400-mg dose level, and mean estimates of elimination half-life ranged from 21.4 to 68.7 hours. After 28 days of once daily AMG 208 dosing, mean unbound trough concentrations ranged from 12.3 to 199 nM, thus exceeding the in vitro IC_50_ estimate against wild-type MET (5.2 nM) at all dose levels. Among patients who received the 300- and 400-mg AMG 208 doses, unbound trough concentrations approximated or slightly exceeded the in vitro IC_50_ against VEGF-R2 (112 nM), which might explain higher increases in mean PIGF levels that were observed at the 400 mg dose level.

AMG 208 showed encouraging antitumor activity in prostate cancer, as suggested by the CR, 2 PRs, and 3 proliferative responses. In a phase 2 study of cabozantinib in prostate cancer, PFS was longer (23.9 versus 5.9 weeks with cabozantinib versus placebo), and 72% of evaluable patients had regression in soft tissue lesions with cabozantinib treatment [15]. It was postulated that cabozantinib's efficacy may be due to the simultaneous inhibition of MET and VEGF, and that the sole targeting of either the MET or VEGF pathways may not be sufficient in this disease. However, recent findings from the phase 3 study in castration-resistant prostate cancer showed no statistically significant improvement in OS with cabozantinib versus prednisone (median OS: 11 versus 9.8 months) [18], indicating that the hypothesis that efficacy in prostate cancer is due to dual inhibition of MET and VEGF may not be justified. AMG 208 has a different target coverage profile than cabozantinib; hence, lack of efficacy with cabozantinib in the prostate cancer setting may not be of relevance to AMG 208. Furthermore, in another study, androgen deprivation was associated with a switch to MET signaling in prostate cancer cells [19]. All 10 patients with prostate cancer in the current study had previous androgen deprivation therapy.

Differences were observed between investigator-assessed and centrally assessed tumor responses; these may be attributable to inadequacies of RECIST 1.0, which considers bone and cystic lesions as nonmeasurable lesions. Some lesions defined as responders in the site reads per RECIST 1.0 were possibly bone or cystic lesions that the investigators considered as measurable lesions. Moreover, different lesions may have been measured in the site and central reads.

Patients selected by MET protein overexpression and *MET* amplification in gastroesophageal cancers [20, 21], *MET* germline mutations in papillary renal cell cancer [17], and chromosome polyploidy in gastric cancer [22] have been associated with response to MET inhibitors. However, in our exploratory and retrospective analysis of MET in the study, no apparent associations between MET expression, amplification, and mutation status were observed. The results suggest that increased levels of MET expression by IHC did not correlate with response to MET inhibition as observed with AMG 208. One consideration is that some patients were treated during dose-escalation of AMG 208 and may not have received adequate doses to inhibit the pathway. Moreover, there may be a minimum threshold at which the MET/CEP7 ratio confers MET dependency and sensitivity to AMG 208, similar to recent reports of HER-2 and trastuzumab in gastric cancer [23]. Finally, these analyses were limited by the small sample size.

AMG 208 at 400 mg was the highest administered dose in this study. Although the MTD was not reached, the 400 mg dose was considered the recommended phase 2 dose. AMG 208 was initially investigated as a MET inhibitor. As mentioned earlier, the responses observed in prostate cancer are likely a result of its multikinase activity, similar to cabozantinib. The study was stopped before enrollment into the dose expansion phase.

It is hypothesized that inhibiting the MET pathway may overcome resistance to various therapies, including anti-EGFR inhibitors, platinum chemotherapy, and radiotherapy [24]. In a phase 1 study, tivantinib, a MET inhibitor and microtubule polymerization inhibitor [25], combined with erlotinib, an EGFR inhibitor, showed promising clinical activity with PR or stable disease observed in 15 of 32 patients [26]; in this same combination study, 6 of 8 patients with NSCLC achieved stable disease [26]. AMG 208 combined with other therapies has yet to be evaluated in clinical studies. Crosstalk between MET and other receptors, such as EGFR, HER2, integrin, and RON, may play a role in the development of resistance to targeted therapies, thus providing rationale to investigate MET pathway inhibitors in combination therapies.

MET continues to be an important target for cancer therapy. In addition to AMG 208, other small molecule MET inhibitors are under clinical development, some being selective inhibitors (eg, volitinib) and others being multikinase inhibitors (eg, crizotinib and cabozantinib) [24]. Several selective small molecule MET inhibitors have recently shown activity in early clinical trials, which include ABT-700 [27], AMG 337 [28], INC280 [29], MSC2156119J [30], SAR125844 [31], and volitinib [32]. Monoclonal antibodies targeting the MET pathway (eg, onartuzumab and rilotumumab) have also been evaluated in clinical trials [24]. Furthermore nanobodies to MET and indozoles have been tested in the preclinical setting as potential inhibitors of MET [33, 34].

In conclusion, AMG 208 was well tolerated as monotherapy at doses up to 400 mg in patients with advanced solid tumors. Future studies evaluating MET pathway inhibitors, particularly in prostate cancer and/or in combination therapies, are warranted.

## MATERIALS AND METHODS

### Eligibility criteria

Eligible patients (≥18 years) had a pathologically documented advanced solid tumor refractory to standard treatment or for which no standard therapy was available. Patients had measurable disease by RECIST version 1.0 [35]. Some patients with prostate cancer and nonmeasurable but evaluable disease (nontarget lesions only) were eligible (modified RECIST 1.0). Patients had an Eastern Cooperative Oncology Group (ECOG) performance status ≤2, life expectancy > 3 months, absolute neutrophil count ≥1.5 × 10^9^/L, platelets ≥100 × 10^9^/L, hemoglobin > 9 g/dL, serum creatinine < 2 mg/dL, calculated creatinine clearance > 60 mL/min, ALT or AST < 3 × the upper limit of normal (ULN; if liver involvement was present, < 5 × ULN), total bilirubin 1.5 × ULN (if liver involvement was present, < 2 × ULN), alkaline phosphatase < 2 × ULN (if liver involvement or bone metastasis was present, ≤5 × ULN), and prothrombin or partial thromboplastin time < 1.5 × institutional ULN. Patients with primary central nervous system tumors or metastases were excluded. Each patient provided informed consent. Institutional review board approval was obtained for all study procedures.

### Study design

This first-in-human, open-label study was to be conducted in two parts: (1) dose escalation and (2) dose expansion. In the dose escalation phase, 3–9 patients were enrolled into 1 of 7 dose cohorts (25, 50, 100, 150, 200, 300, and 400 mg) of AMG 208 (Figure 1). Patients were administered AMG 208 as a single oral dose followed by a 72-hour treatment-free period to evaluate pharmacokinetics. Beginning on day 4, patients received daily oral doses of AMG 208 up to day 28. If no DLT was seen on days 1–28, patients with no evident disease progression received AMG 208.

A DLT was defined as any grade ≥3 nonhematologic or grade 4 hematologic AE occurring during the first 28 days of treatment and possibly AMG 208 related. Treatment-related grade 3 thrombocytopenia could be considered a DLT if accompanied by grade ≥2 hemorrhage. Fatigue (unless grade 3 and lasting > 7 days or grade 4) and lymphopenia were not considered DLTs. For patients with liver involvement or bone metastases, DLTs did not include elevations in alkaline phosphatase unless > 8 × ULN when the baseline level was 2–5 × ULN. For patients with liver involvement, DLTs did not include elevations in AST or ALT unless > 8 × ULN and the baseline level was 3–5 × ULN. Serum creatinine > 2.5 mg/dL was considered a DLT.

In cohorts 1–3, a standard 3+3 design was followed. Enrollment into the next dose level occurred if no patients in the initial cohort experienced a DLT in the first 28 days of treatment. If a DLT occurred, the cohort was expanded to six patients. Enrollment into the next dose level occurred if no DLTs were observed. If ≥2 DLTs were observed among the six patients, enrollment was to be stopped. Following a report of two DLTs (out of six patients) at the 200-mg dose level, the protocol was amended to evaluate an intermediate dose level of 150 mg, and if well tolerated, re-escalation to the 200-mg dose level would occur. In cohorts 4–7, a modified 3+3+3 design was followed in order toprovide additional data to ascertain dose selection; if a second DLT was observed, three additional patients were to be enrolled at the same dose level for a total of at least nine patients. If no DLTs were observed, dose escalation to the next dose level would occur.

To enrich this study, patients with tumors with *MET* amplification or mutation (MET positive) were allowed to enroll into the study at any time at the current dose escalation cohort if a slot was available. If the current escalation cohort was full, eligible MET-positive patients were assigned to the highest dose level deemed safe and well tolerated at the time of enrollment.

### Study objectives

The primary objectives were to evaluate the safety, tolerability, and pharmacokinetics of AMG 208 and determine the MTD. Secondary objectives included evaluating tumor volume changes by CT or magnetic resonance imaging (MRI), decreases in tumor cell proliferation with ^18^F-FLT-PET scanning, potential biomarkers that reflect MET target coverage, and associations among response and MET expression, amplification, and mutation.

### Safety

Safety was evaluated based on AEs, vital signs, clinical laboratory measurements, electrocardiograms, and physical examinations. AEs were graded according to the Common Terminology Criteria for Adverse Events, version 3.0.

### Pharmacokinetics

AMG 208 pharmacokinetics was evaluated after a single dose and after 28 days of repeated daily dose administrations. AMG 208 plasma concentrations were determined by liquid chromatography-tandem mass spectrometry from samples collected predose, 0.5, 1, 2, 4, 8, 24, and 48 or 72 hours after dosing on day 1, and pre-dose, 0.5, 1, 2, 4, 8, 24, and 48 hours on day 28. Pharmacokinetic parameters of observed C_max_, time of C_max_ (t_max_), and AUC_0–24h_ were determined by noncompartmental analysis using Phoenix WinNonlin software version 6.3 (Pharsight^®^, St. Louis, MO). AMG 208 accumulation was estimated as a ratio of AUC_0–24h_ on day 28 relative to day 1 for patients who remained on the study for ≥28 days.

### Pharmacodynamics

Serum samples were collected on days 1 and 2; predose on days 8, 15, 22, 28; on day 64; and every 4 weeks thereafter and were analyzed for soluble MET, HGF, PlGF, VEGF-R2, c-Kit, sFlt-1, VEGF, sCTx, P1NP, and BALP. Pharmacodynamic effects were evaluated using time profiles with the mean ratio to baseline and standard error bars by treatment arms.

Levels of soluble MET, PlGF, VEGF, VEGF-R2, c-Kit, and sFlt-1 were quantified using multiplexed sandwich immunoassays with electrochemiluminescent detection (Meso Scale Discovery, Rockville, MD) following the manufacturer's instructions. Levels of HGF were analyzed using an analyte-specific enzyme-linked immunosorbent assay (ELISA; R&D Systems, Minneapolis, MN) following the manufacturer's instructions and were compared to a standard curve. Samples were prepared and analyzed as previously described [36]. Levels of sCTx were quantified using the Serum Crosslaps ELISA (IDS Nordic, Herlev, Denmark) following the manufacturer's instructions. Levels of P1NP were quantified by radioimmunoassay (RIA) using the UniQ PINP RIA kit following the manufacturer's instructions and were compared to a standard curve (Covance Laboratories). Levels of BALP were quantified using the Access Ostase assay, a one-step immunoenzymatic assay, following the manufacturer's instructions (Beckman Coulter, Indianapolis, IN).

### Antitumor activity

Tumor response was assessed by contrast-enhanced CT or MRI at screening; week 5; week 9; and every 8 weeks thereafter until disease progression and was evaluated by investigators based on modified RECIST version 1.0, which allowed some patients with metastatic prostate cancer and only nontarget bone lesions at screening to be evaluated with bone scans only. Response was also evaluated by an independent central imaging laboratory. Proliferative response was assessed by ^18^F-FLT-PET scans at baseline and week 5. An antiproliferative response was defined as ≥25% reduction in ^18^F-FLT SUV_max_.

### Biomarkers

Archival formalin-fixed, paraffin-embedded (FFPE) tumor samples were analyzed for membrane and cytoplasmic MET expression by IHC at Mosaic Laboratories (Lake Forest, CA). Tumors were sectioned and stained with hematoxylin and eosin to identify regions of high tumor-cell content. IHC was performed using an anti-MET antibody (goat IgG, polyclonal, clone AF276) from R&D Systems. Staining was evaluated by a trained pathologist, and expression was evaluated for cellular localization of staining, intensity, subcellular localization, and percentage of tumor cells staining positive. Staining was evaluated on a semi-quantitative scale, and the percentage of tumor cells staining at the following intensities was recorded: 0 (unstained), 1+ (weak staining), 2+ (moderate staining), and 3+ (strong staining). An H-score was calculated based on the percentage of cells stained at each intensity as follows: (3 × percent cells staining at 3+) + (2 × percent cells staining at 2+) + (1 × percent cells staining at 1+).

Archival FFPE tumor samples were analyzed for *MET* amplification by FISH at Histogenex Laboratories (Antwerp, Belgium). Tumors were pretreated and stained with MET/SE7 Probe kit (KBI-10719, Kreatech, Durham, NC) using a VP2000 autostainer and a Thermobrite Hybrid system (Abbott Molecular, Des Plaines, IL) following the manufacturers' instructions. Twenty nuclei were evaluated per sample, and the quantification of MET and SE7 signals was used to calculate the average MET copies per nuclei, average centromeric copies per nuclei, and the ratio of MET copies to SE7 copies.

DNA was extracted from archival FFPE tumor samples and interrogated for MET mutations using the SURVEYOR Nuclease assay (Transgenomic, Inc., Omaha, NE) followed by Sanger sequencing.

### Statistical analysis

Descriptive statistics on continuous data included means, medians, standard deviations, and ranges. Categorical data were summarized by frequency counts and percentages. All patients who received ≥1 dose of AMG 208 were included in the safety analyses. Treatment-emergent and treatment-related AEs occurring after the initial dose of AMG 208 and before the end of the study or 30 days after the last dose of AMG 208 (whichever occurred later) were presented descriptively. No formal analysis was done for efficacy. An exploratory summary of tumor response was produced. Descriptive analyses for biomarkers and associations between biomarkers and efficacy were produced. Results from the primary analysis (date of data cutoff: December 17, 2012) are presented.
